# Supplementary material for: Conditional chemoconnectomics (cCCTomics) as a strategy for efficient and conditional targeting of chemical transmission
Source: eLife. 2024 Apr 30;12:RP91927. doi: 10.7554/eLife.91927 (PMC11060718; doi:10.7554/eLife.91927)
Supplement: Figure 3—source data 3. [file elife-91927-fig3-data3.zip › Figure 3 source data3 part 1.zip!/Figure 3-Source data 3-part1/1071-1-CRISPResso_Report_osfbjvga.zip!/CRISPResso_on_1071_1/2a.Nucleotide_percentage_quilt.pdf]

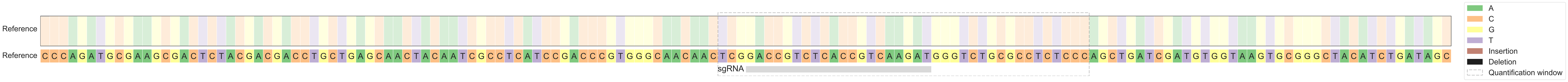
Reference
Reference
C C C A G A T G C G A A G C G A C T C T A C G A C G A C C T G C T G A G C A A C T A C A A T C G C C T C A T C C G A C C C G T G G G C A A C A A C T C G G A C C G T C T C A C C G T C A A G A T G G G T C T G C G C C T C T C C C A G C T G A T C G A T G T G G T A A G T G C G G G C T A C A T C T G A T A G C
sgRNA
A
C
G
T
Insertion
Deletion
Quantification window
